# Data-driven nexus between malaria incidence and World Bank indicators in the Mekong River during 2000–2022

**DOI:** 10.1371/journal.pgph.0003764

**Published:** 2024-09-23

**Authors:** Phuong Hoang Ngoc Nguyen

**Affiliations:** Department of Plant Biotechnology and Biotransformation, Faculty of Biology and Biotechnology, University of Science, Vietnam National University of Ho Chi Minh City (VNUHCM-US), Ho Chi Minh City, Vietnam; Nepal Health Research Council, NEPAL

## Abstract

The increase in hydro dams in the Mekong River amidst the prevalence of multidrug-resistant malaria in Cambodia has raised concerns about global public health. Political conflicts during Covid-19 pandemic led cross-border movements of malaria cases from Myanmar and caused health care burden in Thailand. While previous publications used climatic indicators for predicting mosquito-borne diseases, this research used globally recognizable World Bank indicators to find the most impactful indicators related with malaria and shed light on the predictability of mosquito-borne diseases. The World Bank datasets of the World Development Indicators and Climate Change Knowledge Portal contain 1494 time series indicators. They were stepwise screened by Pearson and Distance correlation. The sets of five and four contain respectively 19 and 149 indicators highly correlated with malaria incidence which were found similarly among five and four GMS countries. Living areas, ages, career, income, technology accessibility, infrastructural facilities, unclean fuel use, tobacco smoking, and health care deficiency have affected malaria incidence. Tonle Sap Lake, the largest freshwater lake in Southeast Asia, could contribute to the larval habitat. Seven groups of indicator topics containing 92 indicators with not-null datapoints were analyzed by regression models, including Multiple Linear, Ridge, Lasso, and Elastic Net models to choose 7 crucial features for malaria prediction via Long Short Time Memory network. The indicator of people using at least basic sanitation services and people practicing open defecation were health factors had most impacts on regression models. Malaria incidence could be predicted by one indicator to reach the optimal mean absolute error which was lower than 10 malaria cases (per 1,000 population at risk) in the Long Short Time Memory model. However, public health crises caused by political problems should be analyzed by political indexes for more precise predictions.

## Introduction

According to a report from the WHO, vector-borne diseases killed more than 700,000 people yearly and accounted for 17% of the deaths caused by all infectious diseases in 2020 [1]. Among mosquito-borne diseases, malaria, a parasitic infection, caused estimated 249 million cases and 608,000 deaths globally in 2022 [2]. Southeast Asia is among the regions impacted most by malaria after Sub-Saharan Africa. Recently, reported cases of multidrug-resistant malaria in Cambodia could spread worldwide rapidly, increasing the risk to public health worldwide [3].

Malaria is caused by the blood parasites *Plasmodium* (*P*. *falciparum*, *P*. *knowlesi*, *P*. *malariae*, *P*. *ovale*, and *P*. *vivax*) through the infected female Anopheles mosquitoes, which can cause symptoms such as fever and shaking. Without treatment, patients can have severe health problems, such as seizures, brain damage, trouble breathing, organ failure, and death [4].

Affected by climate change, approximately one billion people may be exposed to mosquito-borne diseases for the first time by 2080 during extreme global warming [5]. Globally, the number of dengue cases has increased 30-fold in the past 50 years [6]. Some Southeast Asian countries, such as Vietnam, the Philippines, and Malaysia, have been seriously affected by severe dengue epidemics [7]. Malaria situations in GMS were divergent in the past two decades. Myanmar contributed 92.5% of total malaria cases while China was certified as malaria free in 2021 [2].

Furthermore, more than 200 large dams planned, completed, or under construction on the Mekong mainstream and its tributaries have raised concerns about increased mosquito habitats and water-related vector-borne diseases [8, 9]. In addition, international travel and globalization could increase the spread of infectious diseases worldwide [10, 11]. Several severe viruses, such as the Zika virus [12], MERS-CoV, and SARS-CoV-2, have spread far beyond their origin by air travel, causing the COVID-19 pandemic. In Lower Mekong (LM) countries, where the growth rate of tourism was the fastest among Asia-Pacific regions, nearly 60 million tourists visited these regions in 2017 [13]. Multidrug-resistant infectious diseases in that region could be dispersed globally through international tourism and transport [3].

Controlling the spread of infectious diseases requires effective coordination between the health and informational sectors of transnational governments in the Greater Mekong Subregion (GMS). However, several barriers in LM countries, such as information systems based on paper reports, a low level of computerization, and a shortage of health workers with data science training in the field of preventive health care, have been common challenges in addition to their ecological distribution conflicts [14]. These factors have slowed down data sharing for disease surveillance and effective policymaking.

Published studies in the region have shown correlations between mosquito-borne diseases with many sociodemographic and environmental factors. A multivariate analysis of dengue-like diseases in suburban communities in Laos and Thailand revealed that age, education, and occupation were associated with infection rates in suburban Laos and rural Thailand [15]. Many studies in Mekong countries indicate a strong relationship between dengue incidence with temperature and humidity [16, 17]. A study in rubber forests confirmed that industrial rubber plantations provided shelter for mosquitoes and increased the incidence of mosquito-borne diseases [18]. In the Mekong Delta, a waterborne disease index was used to map dengue for a remote sensing study in Vietnam, which revealed clear seasonal variation in dengue fever according to changes in climatic factors [19]. In general, previous studies about factors affecting mosquito-borne diseases in LM were scattered and inadequate because inconsistent data and information have not been shared between countries. In addition, those studies in the region have not employed global open data portals or widely recognizable indicators, e.g., the World Bank indicators, the Air Quality Index, or the Human Development Index. These global indicators are being used in reports by many international organizations and have become references for information exchange worldwide.

Several climatic factors, such as temperature, rainfall, and humidity, have been widely used for time series forecasting via generalized linear models (GLMs), autoregressive integrated moving averages (ARIs), seasonal autoregressive integrated moving averages (SARIMAs), and Holt-Winters models [16]. The GLM assumes a linear relationship between targets and features, while the ARIMA model assumes a linear relationship between past values and current values. This finding is inconsistent with the nonlinear seasonal climatic features in the real world. SARIMA and Holt-Winters can deal with seasonal data better than the former. However, they assume that the data are stationery and deal with stable short-term data better than long-term data. ARIMA, SARIMA, and Holt-Winters are used for univariate data; hence, they lack explanatory power, which provides insights into the underlying factors affecting the models.

Long Short Time Memory (LSTM) is an advanced Recurrent neural network (RNN) that can be used for multivariate sequential data and addressing the vanishing or exploding gradient problem in traditional RNNs [20]. They have been applied for the classification, processing, and prediction of sequential data such as time series, handwriting, and voice data. In GMS, LSTM has been used for predicting malaria in China [21] or dengue in Vietnam [22] by climatic factors as the features.

Using too many features in prediction can cause overfitting and computational inefficiency. Ridge is a regularization technique used for preventing overfitting in linear regression models [23, 24]. It uses L2 regularization, which is controlled by the tuning parameter alpha, to shrink the coefficients toward zero and prevent overfitting, hence resulting in a more robust and accurate model. Similarly, Lasso [25] is another regularization technique that uses L1 regularization to select features, but this approach could cause information loss due to the limited recognizability of collinearity between important indicators and their collinear counterparts. Elastic Net combines both Lasso and Ridge by learning from their shortcomings to improve the regularization.

This research aims to find critically impactful World Development Indicators (WDIs) correlated with malaria incidence in the GMS and used multiple linear regression combined with Ridge, Lasso, and Elastic Net to select features for time series forecasting by LSTM.

## Methods

**Fig 1:**
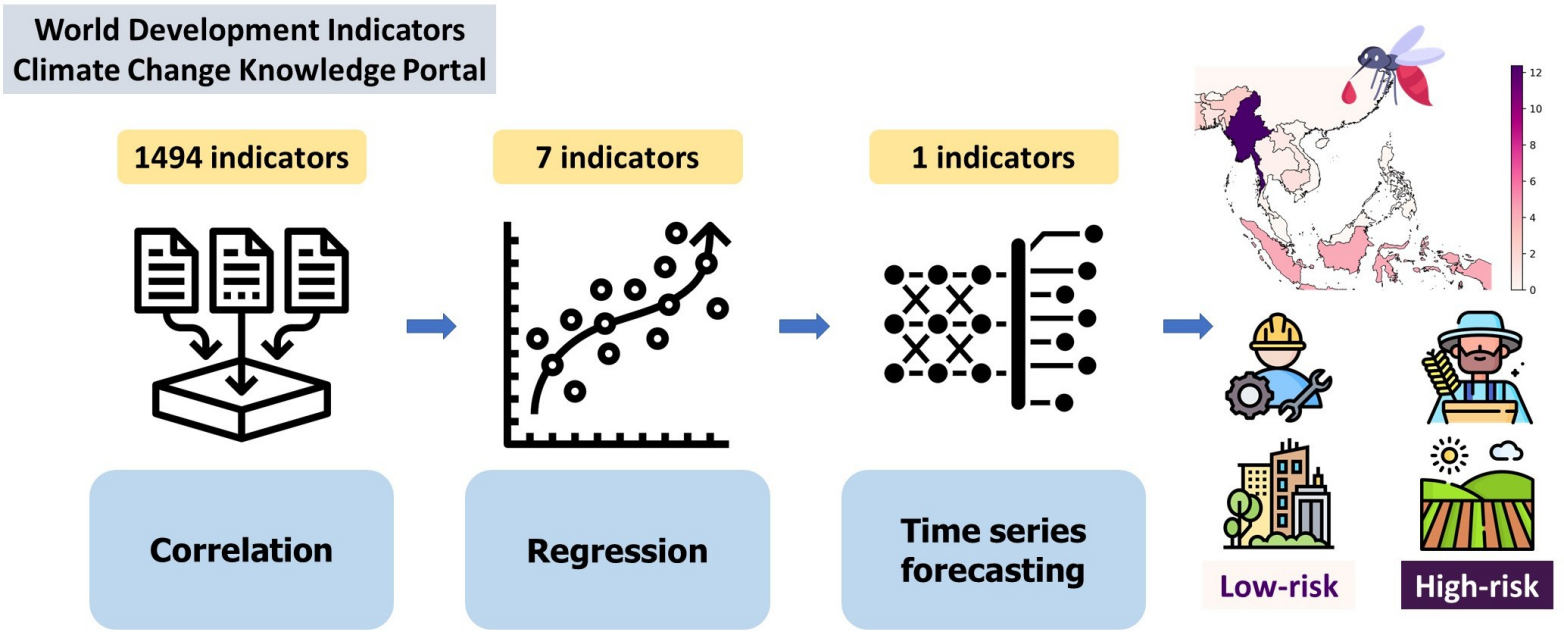
Flowchart of the methodology used in this research.

The methodology used in this work is described in Fig 1, which includes the following steps: data preprocessing, correlational analysis, regression, and time series forecasting.

The World Bank datasets included 1494 time series indicators from 2000–2022 from six countries in the Greater Mekong Subregion. Among the set of indicators strongly correlated with malaria incidence in most countries, seven were selected as the features for Multiple Linear Regression models combined with Ridge, Lasso, and Elastic Net techniques. One indicator critically affecting the linear models was chosen for time series forecasting by Long Short Time Memory Network. The vectors of map were downloaded from public domain https://www.naturalearthdata.com/. The clipart icons were downloaded from public domain https://www.flaticon.com/ including:

https://www.flaticon.com/free-icon/data-collection_2103533

https://www.flaticon.com/free-icon/analysis_1239623

https://www.flaticon.com/free-icon/deep-learning_12031359

https://www.flaticon.com/free-icon/engineer_9321656

https://www.flaticon.com/free-icon/harvest_10132205

https://www.flaticon.com/free-icon/cityscape_2451728

https://www.flaticon.com/free-icon/field_4614470

https://www.flaticon.com/free-icon/malaria_6037989.

### Datasets

The WDI dataset of the updated version (May 30, 2024) for GMS, which included Cambodia, the People’s Republic of China, the Lao People’s Democratic Republic (Lao PDR), Myanmar, Thailand, and Vietnam, was downloaded from the World Bank Group [26]. The dataset contained 1492 time series indicators, including the indicator of malaria incidence per 1000 population at risk (WDI code: SH.MLR.INCD.P3), from 2000 to 2022. In addition, another dataset of climatic indicators containing annual mean average temperature and precipitation in the GMS from 2000 to 2022 was downloaded from the Climate Change Knowledge Portal (CCKP) [27]. The WDI and CCKP datasets were combined into the final dataset containing 1494 indicators from 2000 to 2022 for six countries of the Mekong River. The datasets are provided in S1 Table.

### Correlation

Each yearly observation was treated as a data point. Pearson correlation coefficients (ρ) were calculated by equation (Eq) (1) for two sets of indicators, with x for malaria and y for the other indicators. The indicator of incidence of malaria per 1,000 population at risk (WDI code: SH.MLR.INCD.P3) as written shortly as malaria is provided in S1 Table. $\bar{x}$ and $\bar{y}$ are the sample means of the two arrays of values. A ρ close to +1 or -1 indicates a strong positive or negative correlation, respectively, while a ρ close to 0 indicates no correlation [28].

Distance correlation was calculated by the equation in Eq (2), in which D(x_i_, x_j_) is the Euclidean distance between two sets of indicators, with x for malaria and y for the other indicators [29]. The Distance correlation ranges from 0 to 1, where 0 implies independence between two indicators and 1 implies a linear relationship.

The indicators with high correlation coefficients, > = 0.8 or < = -0.8 for Pearson and > = 0.8 for Distance correlation, which were found similarly between GMS countries, were put into the sets from 1 to 6.

### Regression

The features in each topic or topic group for the regression models were selected from the indicators containing > = 20 datapoints that were strongly correlated with malaria and found similarly in most countries. The MLR models which were established in Eq (3) with malaria (y), the number of other independent variables (k), the k^th^ feature (x_k_), the regression coefficient (weight) of the k^th^ feature (β_k_), and the intercept (β_0_). The datapoint values in each topic or topic group were normalized between 0 and 1. Ridge, Lasso, and Elastic Net algorithms were applied with an alpha range from 10^−5^ to 10^2^ for feature selection in time series forecasting.

### Time series forecasting

The many-to-one architecture of LSTM consists of the following four layers. The 1^st^ LSTM layer takes mini batches in the sliding window from the time series input and returns the whole sequence. The number of neurons in the 1^st^ LSTM layer equals the number of features multiplied by the size of the sliding window. The 2^nd^ LSTM layer with the same number of neurons receives the sequence from the 1^st^ LSTM layer but only returns the same number of features. The next dense layer with the number of neurons is the same as the size of the features. The last dense layer outputs the predicted value. The LSTM setting parameters for the sequential model include normalization between 0 and 1, a training ratio from 0.6 to 0.8, 10 to 30 epochs, a batch size from 10 to 30, and a sliding window size (window size) from 1 to 9.

### Performance metrics for evaluation

The performance metrics for model evaluation were calculated by Eqs (4)–(7). The absolute error (MAE), mean absolute percentage error (MAPE), mean square error (MSE), and coefficient of determination (R2) were used for evaluating the MLR and LSTM models.

The mean absolute error (MAE) in Eq (4) is the average of the absolute differences between the predicted and observed cases for all observations. The mean absolute percentage error (MAPE) in Eq (5) is the average relative error as a percentage of the observed data. The mean squared error (MSE) in Eq (6) is the average squared difference between the predicted malaria case and the observed value. The coefficient of determination (R-squared or R2) in Eq (7) is the proportion of the malaria case that is predictable from the features of the model. In these equations, *y_i_*, ${\hat{y}}_{i},\;{\bar{y}}_{i}$, and *n* are the observed case, the predicted case, the average value of the observed cases, and the total number of observations, respectively.


$$\rho =\frac{\sum_{i=1}^{n}\left(x_{i}-\bar{x})(y_{i}-\bar{y}\right)}{\sqrt{\sum_{i=1}^{n}{\left(x_{i}-\bar{x}\right)}^{2}\sum_{i=1}^{n}{\left(y_{i}-\bar{y}\right)}^{2}}}$$ (1)

$${dCov}_{n}^{2}(x,y)=\frac{1}{n^{2}}\sum_{i=1}^{n}\sum_{j=1}^{n}D(x_{i},x_{j}).D(y_{i},y_{j})$$ (2)

$$y=\beta_{1}x_{1}+\beta_{2}x_{2}+\cdots +\beta_{k}x_{k}+\beta_{0}$$ (3)

$$MAE=\frac{1}{n}\sum_{i=1}^{n}\left|y_{i}-{\hat{y}}_{i}\right|$$ (4)

$$MAPE=\frac{1}{n}\sum_{i=1}^{n}\left|\frac{y_{i}-{\hat{y}}_{i}}{y_{i}}\right|*100$$ (5)

$$MSE=\frac{1}{n}\sum_{i=1}^{n}{\left(y_{i}-{\hat{y}}_{i}\right)}^{2}$$ (6)

$$R^{2}=1-\frac{\sum_{i=1}^{n}{\left(y_{i}-{\hat{y}}_{i}\right)}^{2}}{\sum_{i=1}^{n}{\left(y_{i}-{\bar{y}}_{i}\right)}^{2}}$$ (7)

### Software

The calculation and visualization were implemented in Python 3.9.16 with the following main packages: Cartopy 0.22.0, Geopandas 0.9.0, Geoplot 0.5.1, Keras 2.12.0, Matplotlib 3.7.1, Numpy 1.23.5, Pandas 1.5.3, Scikit-Learn 1.2.1, and TensorFlow 2.12.0.

## Results

### Trends in malaria incidence

**Fig 2:**
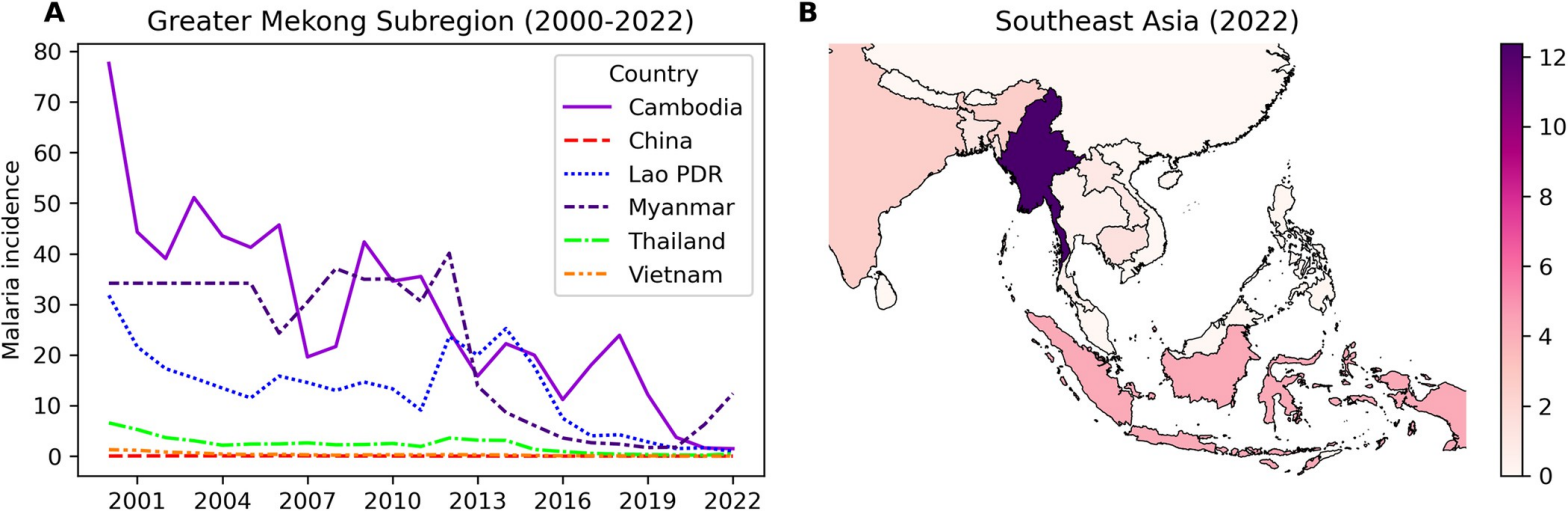
Spatiotemporal overview of the incidence of malaria (cases per 1000 population at risk). (A) The trends in malaria incidence in the Greater Mekong Subregion during 2000–2022. (B) The Greater Mekong Subregion in 2022. The vectors of map were downloaded from public domain https://www.naturalearthdata.com/.

In the GMS, Myanmar, Cambodia, and Laos were among the top countries with the most cases of malaria during 2000–2022 (Fig 2). The spread of a multidrug-resistant co-lineage of *P*. *falciparum* malaria, named KEL1/PLA1, across Cambodia could lead to a peak time of malaria during 2008–2013 [30]. Similar peaks repeated later in Myanmar, Laos, and Thailand. Myanmar contributed 92.4% indigenous malaria cases and 95.0% indigenous *P*. *falciparum* cases in 2021–2022 [2]. The increase of malaria in Myanmar from 78 000 cases in 2019 to 584 000 cases in 2022 amid political instability, which was shown at the end of line plot of Fig 2A, led cross-border movements of individuals to seek health care in Thailand. Nevertheless, the other GMS countries are aiming for certification of malaria elimination like China, which was successfully certified malaria free in 2021.

### Correlation

#### Overview

**Fig 3:**
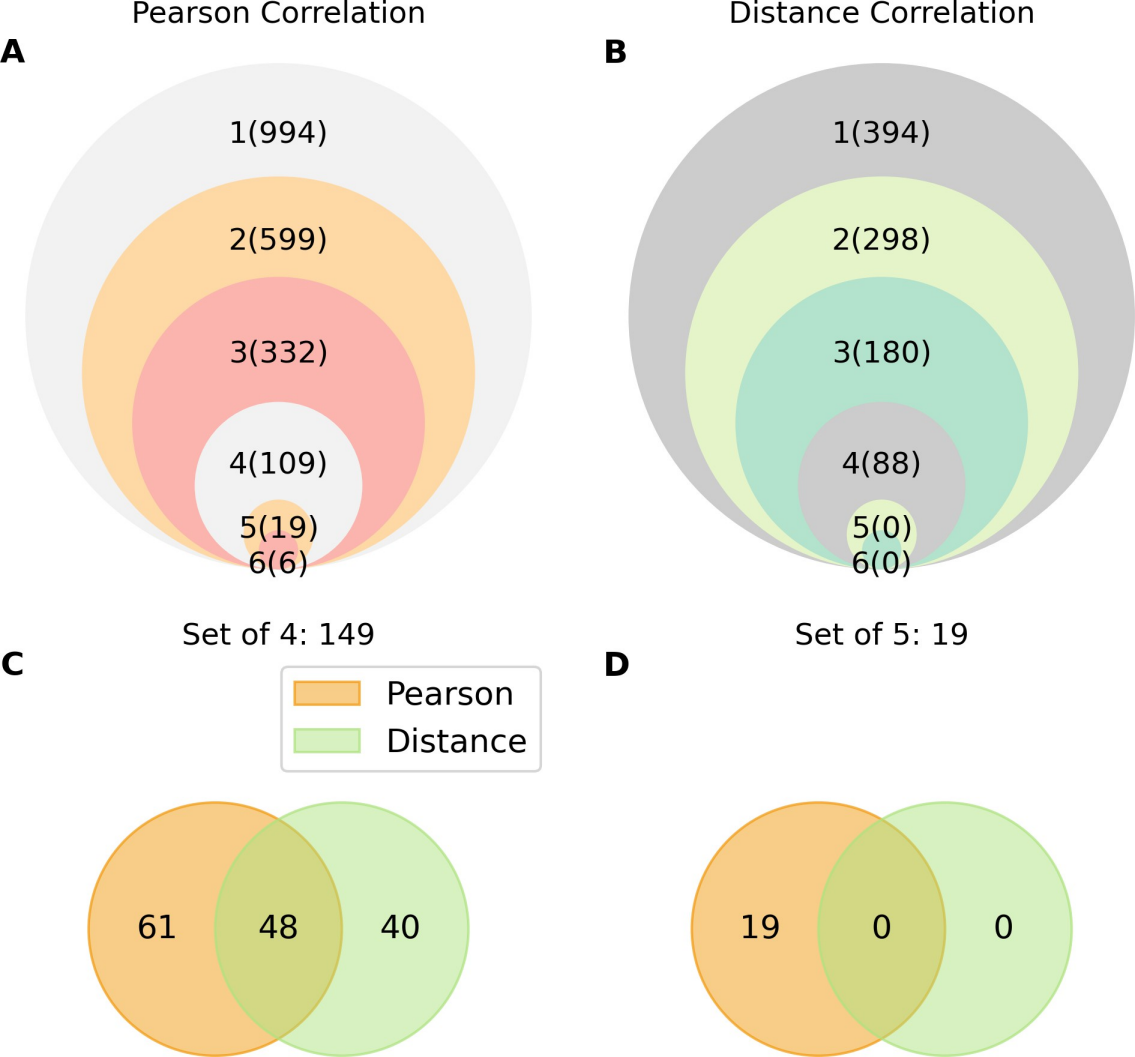
The nested collections of the indicators correlated strongly with malaria according to Pearson and Distance correlation analyses. (A) The number of indicators strongly correlated with malaria incidence according to Pearson correlation analysis; the results were similar for one to six countries, and the values are shown in parentheses. (B) The number of indicators strongly correlated with malaria incidence according to the Distance correlation coefficient, which was similar for one to six countries, with the corresponding indicators in parentheses. (C) The set of 4, containing 149 indicators highly correlated with malaria incidence, was found similarly for four countries with 109 indicators were identified by Pearson correlation, and 88 indicators were identified by Distance correlation, with 48 intersecting indicators. (D) The set of 5, containing 19 indicators highly correlated with malaria incidence, was found similarly for five countries with 19 indicators were identified by Pearson correlation, and 0 indicators were identified by Distance correlation.

Among a total of 1494 combined indicators of WDI and CCKP, 994 and 394 unique indicators, respectively, were strongly correlated with malaria incidence in the GMS countries according to Pearson and Distance correlation (Fig 3).

**Fig 4:**
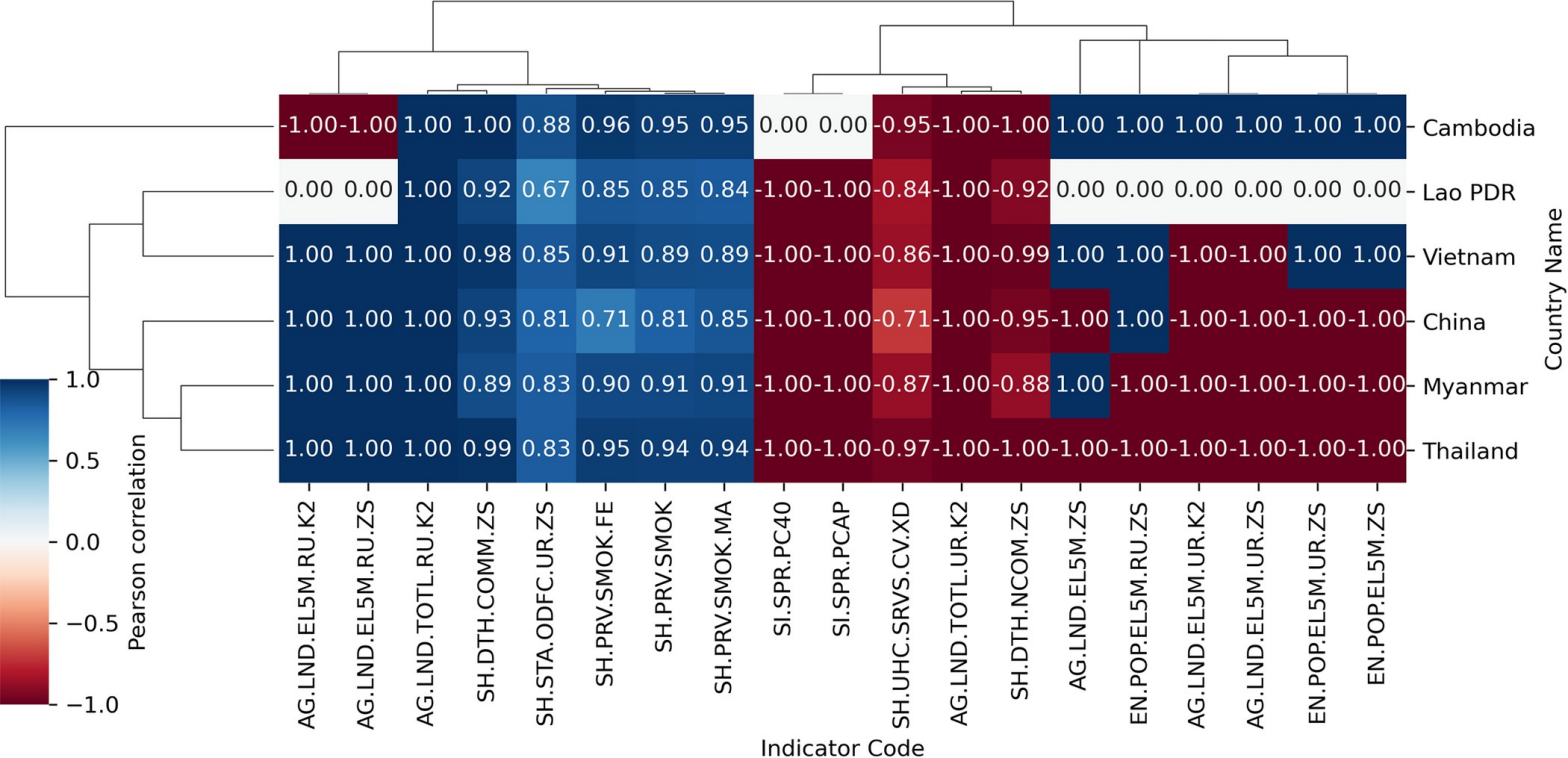
Pearson correlation coefficients of 19 indicators were found for five countries. The linkage for hierarchical clustering uses the complete method and Euclidean metric. Zero (0.00) values contain null values.

Table 1 summarizes the number of indicators in the set of 4 and 5 which were counted by topic groups, number of datapoints, urbanization, and gender. Fig 4 and Table 2 present the Pearson correlation coefficients in the set of 5. The coefficients in the set of 4 are provided in S2 Table.

**Table 1:** Number of indicators in the set of 5 and 4 which were counted by topic groups, number of datapoints, urbanization, and gender.

					Urbanization						Gender					
					R	U	N	R	U	N	F	M	N	F	M	N
	No.	Topic groups | Datapoints	Ab	Bl	Ab	Ab	Ab	Bl	Bl	Bl	Ab	Ab	Ab	Bl	Bl	Bl
Set of 5	1	Environment	0	10	0	0	0	4	4	2	0	0	0	0	0	10
	2	Health	1	7	0	1	0	0	1	6	0	0	1	1	1	5
	3	Poverty	0	2	0	0	0	0	0	2	0	0	0	0	0	2
		Sum	1	19	0	1	0	4	5	10	0	0	1	1	1	17
Set of 4	1	Economic Policy & Debt	17	23	0	0	17	0	0	23	0	0	17	0	0	23
	2	Education	0	2	0	0	0	0	0	2	0	0	0	1	0	1
	3	Environment	6	18	1	4	1	5	10	3	0	0	6	0	0	18
	4	Financial Sector	0	6	0	0	0	0	0	6	0	0	0	1	0	5
	5	Health	50	71	3	3	44	3	3	65	16	13	21	19	15	37
	6	Infrastructure	1	2	0	0	1	0	0	2	0	0	1	0	0	2
	7	Poverty	0	2	0	0	0	0	0	2	0	0	0	0	0	2
	8	Private Sector & Trade	2	7	0	0	2	0	0	7	0	0	2	0	0	7
	9	Public Sector	0	1	0	0	0	0	0	1	0	0	0	0	0	1
	10	Social Protection & Labor	16	17	0	0	16	0	0	17	5	5	6	5	5	7
		Sum	92	149	4	7	81	8	13	128	21	18	53	26	20	103

Datapoints: ‘Ab’: number of yearly datapoints > = 20; ‘Bl’: any number of yearly datapoints. Urbanization: ‘U’: indicator codes and names contain the words of ‘urban, UR, URB’; ‘R’: Indicator codes and names contain the words of ‘rural, RU, and RUR’; ‘N’: indicator codes and names do not contain the words of ‘urban, UR, URB’ or ‘rural, RU, and RUR’. Gender: ‘F’: indicator codes and names contain the words of ‘female, FE’; ‘M’: indicator codes and names contain the words of ‘male, MA’; ‘N’: indicator codes and names do not contain the words of ‘female, FE’ or ‘male, MA’.

**Table 2:** Correlation coefficients of the indicators were found similarly for 5 Greater Mekong Subregion countries.

No.	Indicator Code	Indicator Name	Cambodia	China	Lao PDR	Myanmar	Thailand	Vietnam
1	AG.LND.EL5M.RU.K2	Rural land area where elevation is below 5 meters (sq. km)	-1.00	1.00	NaN	1.00	1.00	1.00
2	AG.LND.EL5M.RU.ZS	Rural land area where elevation is below 5 meters (% of total land area)	-1.00	1.00	NaN	1.00	1.00	1.00
3	AG.LND.EL5M.UR.K2	Urban land area where elevation is below 5 meters (sq. km)	1.00	-1.00	NaN	-1.00	-1.00	-1.00
4	AG.LND.EL5M.UR.ZS	Urban land area where elevation is below 5 meters (% of total land area)	1.00	-1.00	NaN	-1.00	-1.00	-1.00
5	AG.LND.EL5M.ZS	Land area where elevation is below 5 meters (% of total land area)	1.00	-1.00	NaN	1.00	-1.00	1.00
6	AG.LND.TOTL.RU.K2	Rural land area (sq. km)	1.00	1.00	1.00	1.00	1.00	1.00
7	AG.LND.TOTL.UR.K2	Urban land area (sq. km)	-1.00	-1.00	-1.00	-1.00	-1.00	-1.00
8	EN.POP.EL5M.RU.ZS	Rural population living in areas where elevation is below 5 meters (% of total population)	1.00	1.00	NaN	-1.00	-1.00	1.00
9	EN.POP.EL5M.UR.ZS	Urban population living in areas where elevation is below 5 meters (% of total population)	1.00	-1.00	NaN	-1.00	-1.00	1.00
10	EN.POP.EL5M.ZS	Population living in areas where elevation is below 5 meters (% of total population)	1.00	-1.00	NaN	-1.00	-1.00	1.00
11	SH.DTH.COMM.ZS	Cause of death, by communicable diseases and maternal, prenatal and nutrition conditions (% of total)	1.00	0.93	0.92	0.89	0.99	0.98
12	SH.DTH.NCOM.ZS	Cause of death, by non-communicable diseases (% of total)	-1.00	-0.95	-0.92	-0.88	-1.00	-0.99
13	SH.PRV.SMOK	Prevalence of current tobacco use (% of adults)	0.95	0.81	0.85	0.91	0.94	0.89
14	SH.PRV.SMOK.FE	Prevalence of current tobacco use, females (% of female adults)	0.96	0.71	0.85	0.90	0.95	0.91
15	SH.PRV.SMOK.MA	Prevalence of current tobacco use, males (% of male adults)	0.95	0.85	0.84	0.91	0.94	0.89
16	SH.STA.ODFC.UR.ZS	People practicing open defecation, urban (% of urban population)	0.88	0.81	0.67	0.83	0.83	0.85
17	SH.UHC.SRVS.CV.XD	UHC service coverage index	-0.95	-0.71	-0.84	-0.87	-0.97	-0.86
18	SI.SPR.PC40	Survey mean consumption or income per capita, bottom 40% of population (2017 PPP $ per day)	NaN	-1.00	-1.00	-1.00	-1.00	-1.00
19	SI.SPR.PCAP	Survey mean consumption or income per capita, total population (2017 PPP $ per day)	NaN	-1.00	-1.00	-1.00	-1.00	-1.00

Note: NaN: null value.

In the set of 5 containing 19 indicators which were found similarly for five GMS countries, most of them (10/19) belong the topic group of Environment (Table 1). The other topic groups are Health (7/19) and Poverty (2/19). Among 149 indicators in the set of 4, half of them (71/149) belong to the topic group of Health. The second and third leading topic groups are Economic Policy & Debt (23/149) and Social Protection & Labor (17/149). The set of 4 contains 92 indicators with > = 20 datapoints during 2000–2022. However, most of indicators in the set of 5 contain less than 20 datapoints, from two to seven datapoints per country, except the only indicator of people practicing open defecation in urban (SH.STA.ODFC.UR.ZS). The number of datapoints in each indicator is provided in S3 Table.

### Environment & demographics

In the topic group of Environment, the rural land area (AG.LND.TOTL.RU.K2, Fig 4) was perfectly positively correlated with malaria incidence, as opposed to the urban land area (AG.LND.TOTL.UR.K2, Fig 4) in all countries. Similarly, the rural land area where the elevation is less than 5 meters (AG.LND.EL5M.RU._, Fig 4) was perfectly positively correlated with malaria, in contrast to the urban land area where the elevation is less than 5 meters (AG.LND.EL5M.UR._, Fig 4) in all four countries except Cambodia.

Moreover, the population living in areas where the elevation is less than 5 meters (EN.POP.EL5M._, Fig 4) was completely positively correlated with malaria cases in Cambodia and Vietnam as opposed to Myanmar and Thailand. Meanwhile, the population density (EN.POP.DNST, S2 Table), population in largest city (EN.URB.LCTY, S2 Table), and the population in urban agglomerations of more than one million (EN.URB.MCTY.TL.ZS, S2 Table) were strongly negatively correlated with malaria incidence in most countries.

On the other hand, the population ages under 4 (SP.POP.0004., S2 Table), population ages 0–14 (SP.POP.0014., S2 Table), and population ages 10–14 (SP.POP.1004., S2 Table) were correlated strongly positively with malaria incidence, in contrast to the population ages 55–59 (SP.POP.5559.MA.5Y, S2 Table) and population ages above 80 (SP.POP.80UP.MA.5Y, S2 Table).

Income per capita (SI.SPR._, Fig 4), GDP per capita (NY.GDP.PCAP._, S2 Table), wage and salaried workers (SL.EMP.WORK._, S2 Table), and coverage of social protection & labor programs (per_allsp.cov_pop_tot, S2 Table) were strongly negatively correlated with malaria incidence. In contrast, employment in agriculture (SL.AGR.EMPL._, S2 Table) was strongly positively correlated with malaria incidence, which was like self-employed (SL.EMP.SELF._, S2 Table), and vulnerable employment (SL.EMP.VULN._, S2 Table).

The indicators of environment and demographics suggest living areas, ages, careers, income, social securities have affected malaria incidence.

### Economics

Manufacturing (NV.IND.MANF.CN, S2 Table), industry (NV.IND.TOTL._, S2 Table), service (NV.SRV.TOTL._, S2 Table), merchandise imports from low- and middle-income economies (TM.VAL.MRCH._, S2 Table), researchers in R&D (SP.POP.SCIE.RD.P6, S2 Table) and statistical performance indicators (IQ.SPI.PIL1, S2 Table) were strongly negatively correlated with malaria incidence.

Similarly, individuals using the Internet (IT.NET.USER.ZS, S2 Table), account ownership at a financial institution or with a mobile-money-service provider (FX.OWN.TOTL._, S2 Table), accessibility to clean fuels and technologies for cooking (EG.CFT.ACCS._, S2 Table), and average time to clear exports through customs (IC.CUS.DURS.EX, S2 Table) were strongly negatively correlated with malaria cases, in contrast to power outages in firms in a typical month (IC.ELC.OUTG, S2 Table).

The indicators of economics suggest economic improvement, technology accessibility, infrastructural facilities of information, transportation, and energy help to decrease malaria incidence.

### Health

In the topic groups of health, the cause of death by communicable diseases and maternal, prenatal and nutrition conditions (SH.DTH.COMM.ZS, Fig 4) was strongly positively with malaria incidence, which was similar to infant deaths (SH.DTH.IMRT, S2 Table), neonatal deaths (SH.DYN.NMRT, S2 Table), under-five deaths (.SH.DYN.MORT._., S2 Table), and incidence of HIV (SH.HIV.INCD._, S2 Table). Besides, prevalence of current tobacco use (SH.PRV.SMOK._, Fig 4) was also strongly positively correlated with malaria incidence, which was like people practicing open defecation (SH.STA.ODFC._, Fig 4).

In contrast, universal health coverage (UHC) index (SH.UHC.SRVS.CV.XD, Fig 4) was strongly negatively correlated with malaria incidence, which was similar to people using at least basic drinking water services (SH.H2O.BASW._, S2 Table), people using at least basic sanitation services (SH.STA.BASS._, S2 Table), people with basic handwashing facilities including soap and water (SH.STA.HYGN.ZS, S2 Table).

The indicators of health suggest vulnerable dependent population, health safety and habits have affected malaria incidence.

### Others

In the suspected indicators which were previously reported about the correlation with malaria (Table 3), forest area (AG.LND.FRST._) was associated with malaria incidence in some countries. Climatic indicators of precipitation (PR.) and temperature (TAS.) were uncorrelated with malaria incidence in all countries, which was like air transport (IS.AIR._) in both freight and passenger. Meanwhile, the renewable internal freshwater resources per capita (ER.H2O.INTR.PC) were moderately positively correlated with malaria incidence, especially strong in Cambodia and Vietnam.

**Table 3:** Pearson correlation coefficients between malaria incidence and the suspected indicators.

No.	Indicator Code	Indicator Name	Cambodia	China	Lao PDR	Myanmar	Thailand	Vietnam
1	AG.LND.FRST.K2	Forest area (sq. km)	0.78	-0.78	0.64	0.83	-0.64	-0.86
2	AG.LND.FRST.ZS	Forest area (% of land area)	0.78	-0.78	0.64	0.83	-0.64	-0.85
3	ER.H2O.INTR.PC	Renewable internal freshwater resources per capita (cubic meters)	0.85	0.77	0.61	0.83	0.80	0.85
4	IS.AIR.DPRT	Air transport, registered carrier departures worldwide	-0.38	-0.78	0.03	-0.74	-0.56	-0.67
5	IS.AIR.GOOD.MT.K1	Air transport, freight (million ton-km)	0.34	-0.81	0.14	-0.47	0.09	-0.70
6	IS.AIR.PSGR	Air transport, passengers carried	-0.48	-0.77	-0.04	-0.69	-0.52	-0.64
7	PR	Annual mean precipitation (mm)	-0.11	-0.45	0.05	0.10	-0.01	0.12
8	TAS	Annual mean temperature (°C)	-0.41	0.02	-0.22	-0.31	-0.43	-0.32

### Multiple linear regression model

Although most of the 19 indicators in the set of 5 show a perfect correlation with malaria incidence, they contain very few observations for building dependable models except the indicator of people practicing open defecation in urban (SH.STA.ODFC.UR.ZS). Therefore, 92 indicators containing > = 20 datapoints were considered as the features for regression models from the set of 4. There was high collinearity between these indicators via Pearson correlational analysis as shown in S4 Table. S5 Table provided MLR coefficients when using all indicators in each topic group for building MLR models to predict malaria incidence.

**Fig 5:**
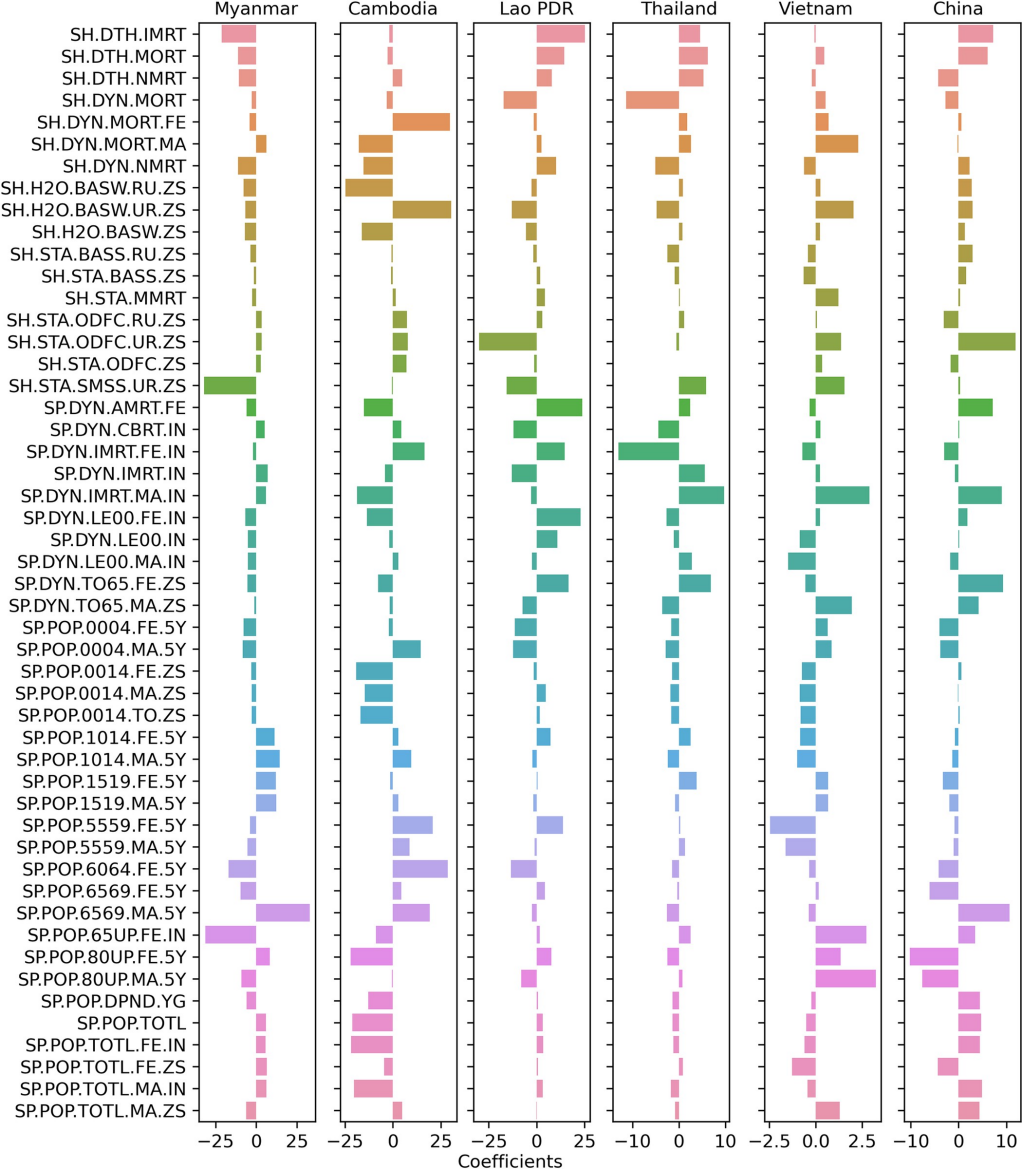
Multiple linear regression coefficients with 50 indicators of the topic group of Health as model features.

When using 50 indicators from the group topic of Health for building MLR models (Fig 5 and S5 Table), each country, each locality (urban and rural regions), and each gender reveals different patterns. The MLR coefficient of people practicing open defecation in urban (SH.STA.ODFC.UR.ZS) in Lao PDR is negative while they were positive in the other countries, especially high in China. However, the MLR coefficient of people practicing open defecation in rural is negative in China while they were positive in the others. In Myanmar, two out-standing indicators with negative coefficients including people using safely managed sanitation services in urban (SH.STA.SMSS.UR.ZS) and population ages 65 and above in female (SP.POP.65UP.FE.IN) while population ages 65–69 in male (SP.POP.6569.MA.5Y) had an out-standing positive coefficient. Nevertheless, the coefficients of population ages 80 and above (SP.POP.80UP._) are noticeably positively high in Vietnam.

Using 50 features such as the indicators from topic group of Health at the same time for MLR regression could cause overfitting models but using too few features such as one or two indicators from the topic group of Infrastructure or Private Sector & Trade could cause under fitting models. Therefore, 92 indicators were divided into 7 groups based on their topics or topic groups for feature selection by MLR, Ridge, Lasso, and Elastic Net regressions. The MLR, Ridge, Lasso, and Elastic Net coefficients for each group are provided in S6 Table. The more the alpha increases in the paths of the regularized coefficients, the coefficients of the more affecting indicators in the models tended more slowly to zero.

**Fig 6:**
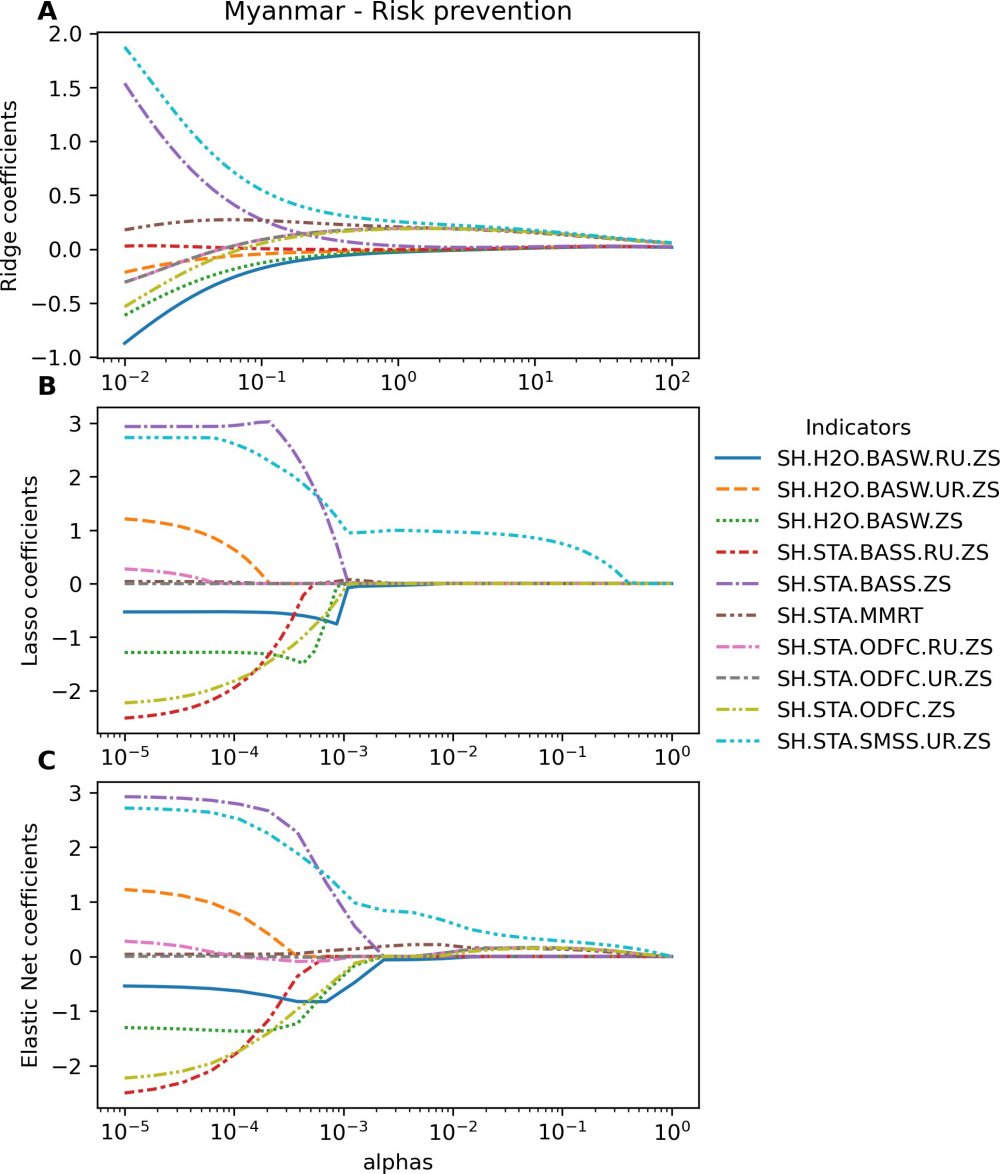
The regularized coefficients of Ridge, Lasso, and Elastic Net in Myanmar using 10 indicators of the group 1—health: Disease prevention, risk factors, and reproductive health. (A) Ridge. (B) Lasso. (C) Elastic Net.

**Fig 7:**
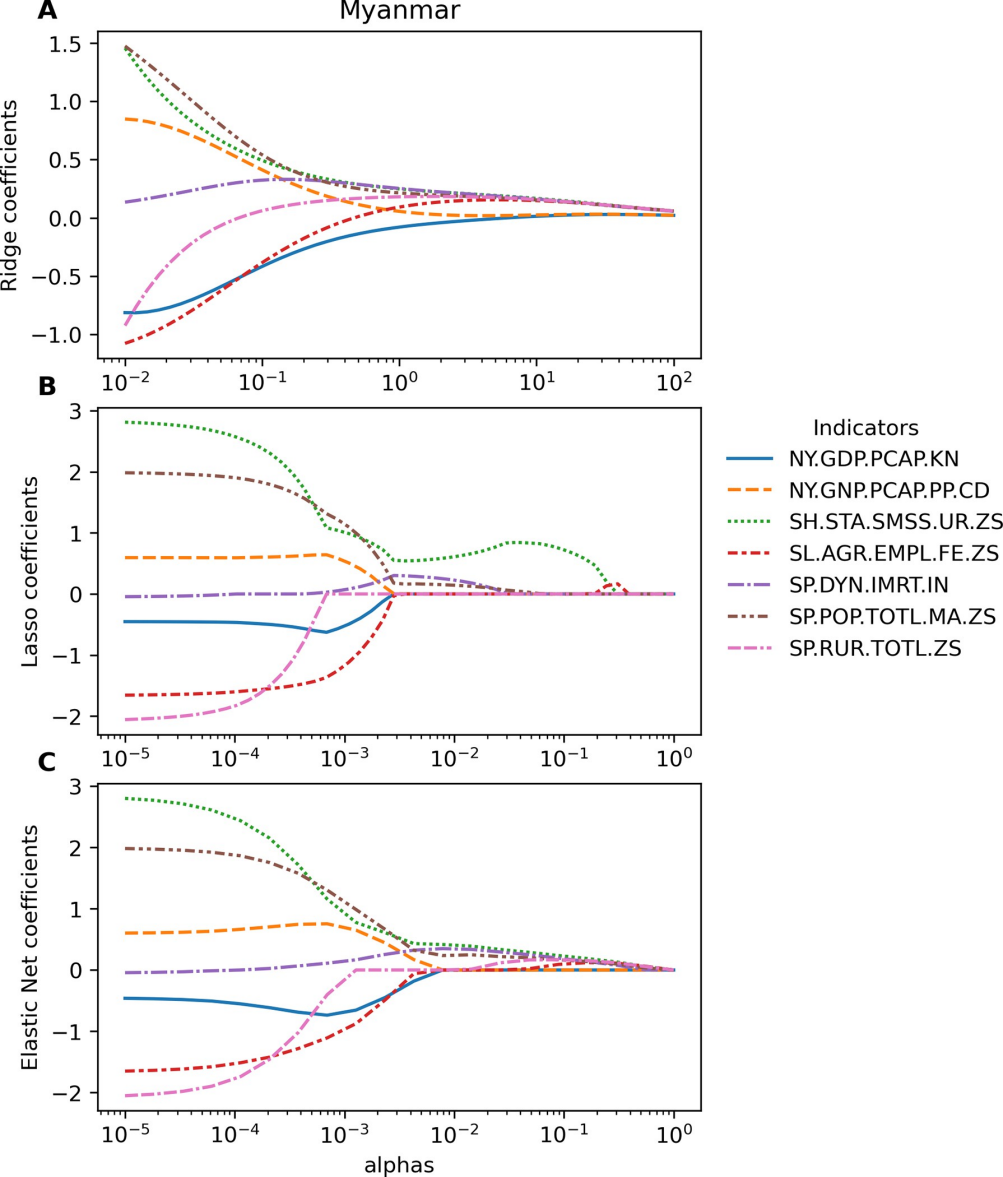
The regularized coefficients of Ridge, Lasso, and Elastic Net in Myanmar using 7 representative indicators which were selected from each group. (A) Ridge. (B) Lasso. (C) Elastic Net.

Table 4 presents the most impacting indicator per each group in each country. The indicator SH.STA.ODFC.RU.ZS was the most impacting feature of the group 1 of Health in most countries, except Myanmar. In Myanmar, SH.STA.SMSS.UR.ZS was the most impacting feature in the group 1 of Health (Fig 6). Moreover, it was also the most impacting feature among seven representative indicators selected from 7 groups in Myanmar (Fig 7).

**Table 4:** The most impacting features selected by Elastic Net regression per each group in each country.

No. Group	1	2	3	4	5	6	7
Topic groups: Topics	Health: Disease prevention, Risk factors, Reproductive health	Health: Mortality	Health: Population: Dynamics, Structure	Economic Policy & Debt: National accounts	Economic Policy & Debt: Official development assistance, Purchasing power parity, Adjusted savings & income	Social Protection & Labor	Environment + Private Sector & Trade
Number of indicators per group	10	16	24	9	8	16	9
Myanmar	SH.STA.SMSS.UR.ZS	SP.DYN.IMRT.IN	SP.POP.TOTL.MA.ZS	NY.GDP.PCAP.KD	NY.GNP.PCAP.PP.CD	SL.AGR.EMPL.FE.ZS	SP.RUR.TOTL.ZS
Cambodia	SH.STA.ODFC.RU.ZS	SH.DYN.NMRT	SP.POP.1519.FE.5Y	NY.GDP.PCAP.KD	DC.DAC.USAL.CD	SL.EMP.VULN.MA.ZS	SP.RUR.TOTL.ZS
Lao PDR	SH.STA.ODFC.RU.ZS	SH.DTH.NMRT	SP.POP.1519.FE.5Y	NY.GDP.PCAP.KD	NY.GDP.PCAP.PP.KD	SL.EMP.SELF.FE.ZS	SP.RUR.TOTL.ZS
Thailand	SH.STA.ODFC.UR.ZS	SP.DYN.AMRT.FE	SP.POP.TOTL.MA.ZS	NV.IND.TOTL.KD	NY.ADJ.DKAP.GN.ZS	SL.EMP.SELF.MA.ZS	SP.RUR.TOTL.ZS
Vietnam	SH.STA.ODFC.RU.ZS	SP.DYN.AMRT.FE	SP.DYN.CBRT.IN	NY.GDP.PCAP.KD	NY.ADJ.DKAP.GN.ZS	SL.AGR.EMPL.FE.ZS	SP.RUR.TOTL.ZS
China	SH.STA.ODFC.UR.ZS	SH.DTH.IMRT	SP.POP.1519.FE.5Y	NV.IND.TOTL.KN	DC.DAC.USAL.CD	SL.AGR.EMPL.ZS	SP.RUR.TOTL.ZS

Note: The coefficients of MLR, Ridge, Lasso, Elastic Net regression are provided in S6 Table.

### Time series forecasting

**Fig 8:**
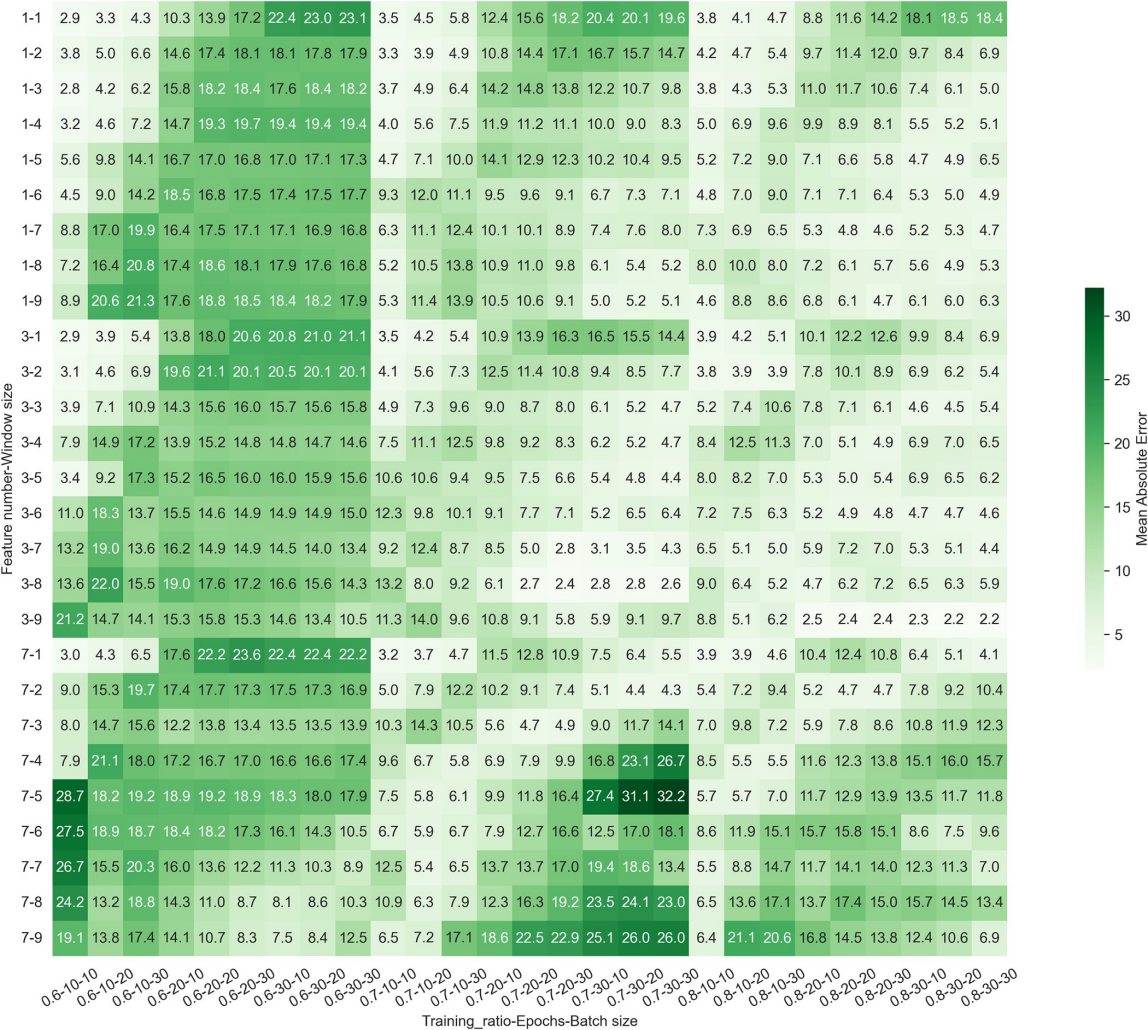
Optimization of LSTM in Myanmar. The parameters of the LSTM model included the number of features, window size, training ratio, epochs, and batch size. The evaluation data of the LSTM network are provided in S7 Table. One-feature models used each of the indicators ’SH.STA.SMSS.UR.ZS’, ’SP.DYN.IMRT.IN’, and ’SP.POP.TOTL.MA.ZS’. Three-feature model used all of three indicators ’SH.STA.SMSS.UR.ZS’, ’SP.DYN.IMRT.IN’, and ’SP.POP.TOTL.MA.ZS’. Seven-feature model used all of seven indicators ’SH.STA.SMSS.UR.ZS’, ’SP.DYN.IMRT.IN’, ’SP.POP.TOTL.MA.ZS’, ’NY.GDP.PCAP.KD’, ’NY.GNP.PCAP.PP.CD’, ’SL.AGR.EMPL.FE.ZS’, and ’SP.RUR.TOTL.ZS’.

**Fig 9:**
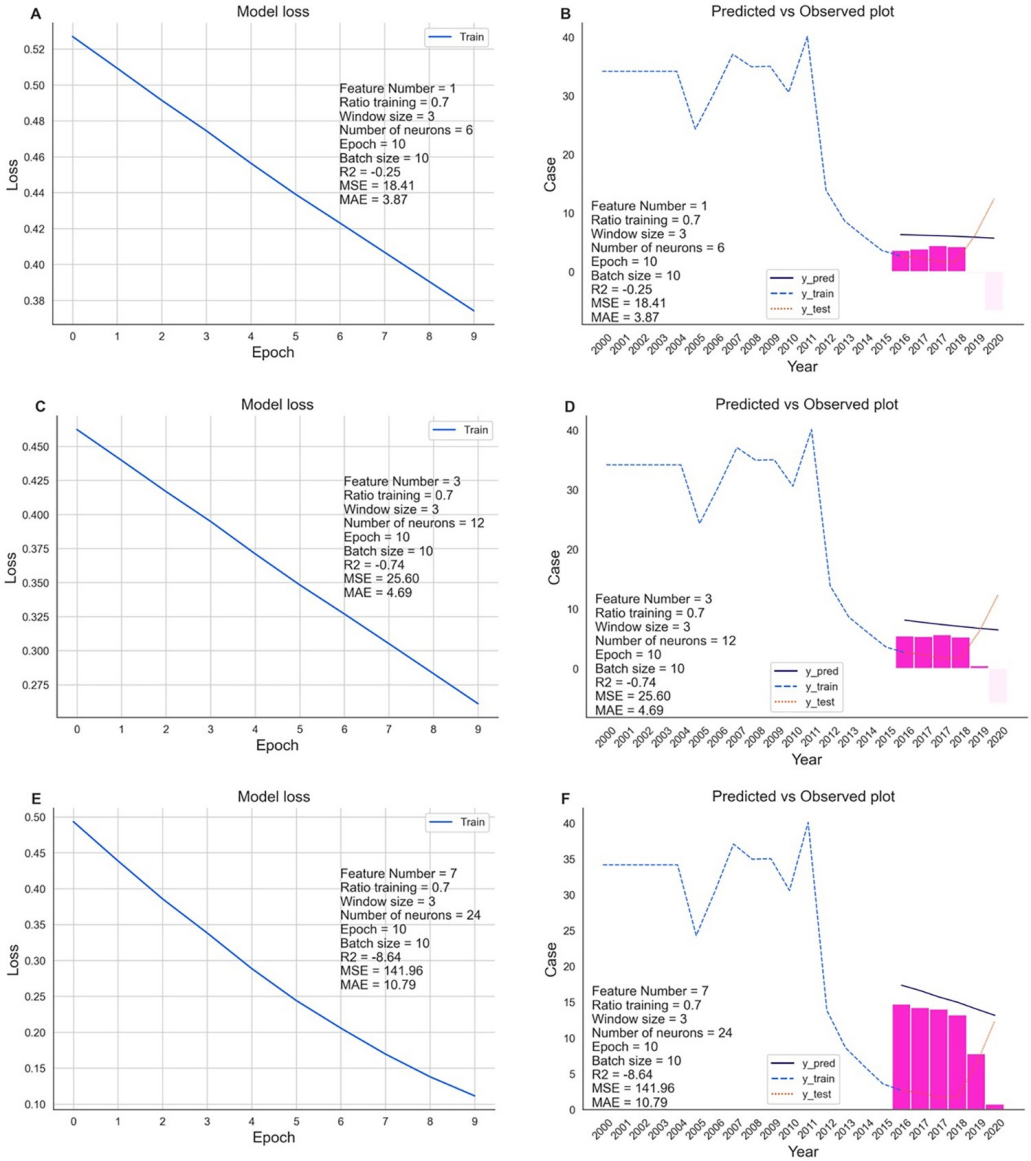
Prediction of malaria cases in Myanmar by the LSTM network with the indicator. (A & B) One-feature model used the indicators ’SH.STA.SMSS.UR.ZS’. (C & D) Three-feature model used all of three indicators ’SH.STA.SMSS.UR.ZS’, ’SP.DYN.IMRT.IN’, and ’SP.POP.TOTL.MA.ZS’. (E & F) Seven-feature model used all of seven indicators ’SH.STA.SMSS.UR.ZS’, ’SP.DYN.IMRT.IN’, ’SP.POP.TOTL.MA.ZS’, ’NY.GDP.PCAP.KD’, ’NY.GNP.PCAP.PP.CD’, ’SL.AGR.EMPL.FE.ZS’, and ’SP.RUR.TOTL.ZS’.

The number of features, training ratio, and window size played significant roles in tuning the optimal models (Fig 8). The MAE values range from 2–32 (mean = 11, median = 10). The lower MAE values indicate the better models. The training ratio 0.6 releases MAE values larger than the models with training ratio 0.7 and 0.8. The window sizes which were lower than 3 or higher than 4 at big epochs and batches also result the higher MAE values. There was no significant difference between the one-feature model (Fig 9A & 9B) and three-feature model (Fig 9C & 9D). Both feature sets were better than the 7 feature sets (Fig 9E & 9F). In general, the predictability of malaria incidence could be predicted by World Bank indicators via machine learning and artificial intelligence at national or subregion levels yearly.

## Discussion

Many previous publications have shown that climatic factors such as temperature, moisture, and precipitation are strongly correlated with mosquito-borne diseases [31–33]. However, this study showed that climatic factors were uncorrelated with malaria incidence in the GMS during 2000–2022. The reason could be that the input data in previous studies were collected locally where latitude, longitude, elevation, and weather changed in limited regions compared to a very wide range of climatic patterns across the different regions of the GMS in this research. This provides hope for local communities in hot and humid regions to combat against not only malaria but also mosquito-borne diseases by considering and affecting other key factors.

Other reports have indicated that many sociodemographic factors, such as urbanization, education, and occupation are strongly correlated with mosquito-borne diseases [15, 34]. This research also reinforces these findings and provides other noticeable indicators. Here, people using at least basic sanitation services and people practicing open defecation are among the crucial indicators affecting regression models. Many previous studies have shown that poor sanitation, open defecation, and improper wastewater management are ideal breeding conditions for mosquitoes [35–38]. Although rapid urbanization in developing countries could cause urban health problems such as air pollution, garbage, heat, the urban island effect, and water containers for larval habitats [39], the results here show that rural land area and rural population living in area where elevation is below 5 meters were correlated positively strongly with malaria incidence, as opposed to urban ones in most GMS countries. However, the limited number of datapoints in land area and population should be considered to conclude urbanization or ruralization facilitates malaria. This pattern was reversed in Cambodia. Tonle Sap Lake, the largest freshwater lake in Southeast Asia [40], could contribute greatly to the larval habitat in Cambodia. This research also revealed that the renewable internal freshwater resources per capita were correlated positively strongly with malaria incidence in most of the GMS, especially in Cambodia and Vietnam. The increase in hydro dams in the Mekong River to satisfy the unstoppable demands of economic development will be one of the main roots for spreading mosquito borne diseases in future.

Furthermore, behaviors at the household level, for example, improving housing quality and removing larval habitats, provided evidences for preventing mosquito-borne diseases [41–43]. A prior research on socioeconomic and household risk factors with malaria showed using wood and dung cakes as cooking fuel were significantly more at risk to have malaria cases [44]. Others reported carbon dioxide as a mosquito attractant on *Aedes* [45, 46], *Culex* [47] and *Anopheles* [48, 49]. Here, the indicators of accessibility of clean fuels and technologies for cooking is correlated negatively strongly with malaria incidence in most GMS countries. Smartphone geospatial apps and other mobile technology-tools have been used for disease surveillance in community [50]. This research also shows that individuals using the Internet is correlated negatively strongly with malaria incidence.

In addition, nutritional conditions, and healthy habits are also among the important key factors for reducing mosquito-borne diseases according to this study. Mosquitoes are attracted by several specific chemicals, such as carbon dioxide, lactic acid, and oct-3-enol, that are emitted by tobacco smokers [45, 46, 51–55]. Here, the prevalence of current tobacco use was also strongly correlated with the incidence of malaria in all GMS countries. In addition to tobacco users, pregnant women who exhale more carbon dioxide also attract more mosquitoes [56]. According to this research, female individuals aged 15–19 years are among most affecting features in regression models. Some odorous compounds produced by skin bacteria and emitted strongly by young adults that might attract mosquitoes [57, 58]. Beside young adults, dependent population such as elderly individuals and infants who usually have weak immune systems and limited mobility were critically affected by malaria.

While most previous reports about predicting malaria and other mosquito-borne diseases by regression and time series forecasting models used climatic factors as features [13, 14, 18, 19, 41], the results here provide another approach using globally referenceable World Bank indicators. Some indicators, such as land area, have changed little for a long time, so it is difficult and expensive to collect data yearly. Although the yearly datasets in specified countries cause a challenge in building high resolution models for certain geographic locality, the recommended features used for building predicting models are among the indicators that are strongly correlated with malaria and are found similarly in most Mekong countries. Those results will deliver stakeholders and policymakers important references to make national and transnational decisions. However, political upheaval and humanitarian crisis which were main reasons of malaria increasing in Myanmar since 2021 have not been estimated by WDI.

Despite many steady efforts towards malaria elimination in GM, antimalarial drug resistance is still a concern in GMS. Malaria is among the most common fatal vector-borne diseases, especially in low- and middle-income countries. LM has been thriving in tourism, a green industry, owing to its diversified original culture and nature, which are less impacted by humans even though this also increases the risk of the spread of communicable diseases. To pursue sustainable development goals, transnational governments in GMS need to communicate effectively by sharing electronic data and information about communicable diseases, including malaria.

## Conclusion

Malaria is still one of the most severe public health problem midst the multidrug-resistant malaria in Cambodia and the increase in hydro dams in the Mekong River. From 1494 indicators from WDI and CCKP, this research provided the sets of 4 and 5 containing respectively 19 and 149 indicators highly correlated with malaria incidence which was found respectively similarly for five and four GMS countries by Pearson and Distance correlation. They indicate malaria incidence are correlated with the living areas, ages, careers, health habits, economic status, technology accessibility, infrastructural facilities of information, transportation, and energy. From the set of 4, 92 indicators containing > = 20 datapoints were analyzed by MLR, Ridge, Lasso, and Elastic Net regressions. Seven most impacting features from seven topic groups were chosen for LSTM model. WDI can be used for predicting malaria incidence by LSTM model with one feature. While WDI could be referred for transnational or national level decisions, certain geographic areas still need high resolution time series indicators such as climatic indicators for disease surveillance. However, public health crises in GMS caused by political instability should be analyzed by political indexes for more precise prediction.

## Supporting information

S1 Table Dataset of world development indicator. (XLSX)

S2 Table Peason and Distance correlation coefficients. (XLSX)

S3 Table Number of datapoints in the set of 4 and 5. (XLSX)

S4 Table Collinearity via Pearson correlation between the indicators of the set of 4. (XLSX)

S5 Table MLR coefficients of total of not-null indicators in the set of 4. (XLSX)

S6 Table MLR, Ridge, Lasso, and Elastic Net coefficients of the indicators in each group of topics. (XLSX)

S7 Table The evaluation and coefficients of long short time memory network for Myanmar. (XLSX)
